# The Course of Serum Inflammatory Biomarkers Following Whiplash Injury and Their Relationship to Sensory and Muscle Measures: a Longitudinal Cohort Study

**DOI:** 10.1371/journal.pone.0077903

**Published:** 2013-10-17

**Authors:** Michele Sterling, James M. Elliott, Peter J. Cabot

**Affiliations:** 1 Centre of National Research on Disability and Rehabilitation Medicine (CONROD), The University of Queensland, Brisbane, Queensland, Australia; 2 Department of Physical Therapy and Human Movement Sciences, Feinberg School of Medicine, Northwestern University, Chicago, Illinois, United States of America; 3 The School of Pharmacy, Pharmacy Australia Centre of Excellence, The University of Brisbane, Queensland, Queensland, Australia; University of Leicester, United Kingdom

## Abstract

Tissue damage or pathological alterations are not detectable in the majority of people with whiplash associated disorders (WAD). Widespread hyperalgisa, morphological muscle changes and psychological distress are common features of WAD. However little is known about the presence of inflammation and its association with symptom persistence or the clinical presentation of WAD. This study aimed to prospectively investigate changes in serum inflammatory biomarker levels from the acute (<3 weeks) to chronic (>3 months) stages of whiplash injury. It also aimed to determine relationships between biomarker levels and hyperalgesia, fatty muscle infiltrates of the cervical extensors identified on MRI and psychological factors. 40 volunteers with acute WAD and 18 healthy controls participated. Participants with WAD were classified at 3 months as recovered/mild disability or having moderate/severe disability using the Neck Disability Index. At baseline both WAD groups showed elevated serum levels of CRP but by 3 months levels remained elevated only in the moderate/severe group. The recovered/mild disability WAD group had higher levels of TNF-α at both time points than both the moderate/severe WAD group and healthy controls. There were no differences found in serum IL-1β. Moderate relationships were found between hyperalgesia and CRP at both time points and between hyperalgesia and IL-1β 3 months post injury. There was a moderate negative correlation between TNF-α and amount of fatty muscle infiltrate and pain intensity at 3 months. Only a weak relationship was found between CRP and pain catastrophising and no relationship between biomarker levels and posttraumatic stress symptoms. The results of the study indicate that inflammatory biomarkers may play a role in outcomes following whiplash injury as well as being associated with hyperalgesia and fatty muscle infiltrate in the cervical extensors.

## Introduction

Whiplash associated disorders (WAD) are a common and costly health problem for western society. Many (up to 50%) of those injured transition to chronicity [1] and current management approaches for both acute and chronic WAD are only modestly effective[2,3]. Further understanding of processes underlying ongoing pain and disability following whiplash injury may facilitate new directions for management of this condition and improve health outcomes. 

Tissue damage or a specific anatomical lesion is not detectable in the majority of patients with WAD[4]. However convergent data from cadaveric, experimental and clinical studies provides evidence supporting the likely presence of tissue damage, particularly involving the zygaophyseal joint[4]. Following injury, proinflammatory cytokines such as IL-1β and TNF-α, produced by most injured cells and activated immune cells play roles in phagocyte proliferation and activation, adhesion and angiogenesis[5]. CRP (C-reactive protein) is an acute-phase reactant released by liver cells and is a marker of underlying low-grade inflammation [6]. Despite the traumatic nature of the whiplash event, the involvement of inflammatory processes has not been well investigated. In a small study (n=11), Kivioja et al [7] showed elevated numbers of cytokine releasing immune cells at 3 days post whiplash injury which was not evident at 14 days, suggesting an initial but resolving inflammatory response. However this study did not explore differences between recovered and non-recovered participants or between those with greater or lesser symptoms. 

Later findings indicate that such differentiations are important. The presence of features such as widespread hyperalgesia, stress related symptoms and pain catastrophising have been shown to be predictive of poor functional recovery [8,9] as well as associated with higher reported pain and disability in the early acute post injury stage [10]. The presence of widespread hyperalgesia has been shown to be a consequence of inflammatory processes [11];, stress exposure can lead to the release of cytokines signalling infection or inflammation [12] and relationships between catastrophising and inflammation have been found [13]. The co-occurrence of hyperalgesia and stress related responses/ symptoms in whiplash injured people with poor recovery suggest that investigation of inflammatory processes is warranted.

Whilst the identification of specific injured tissues has proven to be elusive in WAD, recent investigation has consistently demonstrated the presence of morphological muscle changes in the form of fatty infiltrate in the cervical spine muscles of patients with chronic WAD [14,15]. We have also shown that these fatty infiltrates are present within 4-12 weeks post injury but importantly are significantly greater in participants who develop chronic pain and disability [16]. Although the mechanisms underlying the fatty deposits are not known, circulating inflammatory biomarkers may contribute to degenerative muscle changes [17,18]. 

The aims of this study were to 1) prospectively investigate changes in inflammatory biomarker levels from within 3 weeks of injury to 3 months post injury; 2) to investigate the differences in inflammatory biomarker levels between fully/partially recovered participants and those who report persistent moderate to severe pain related disability at 3 months; 3) to explore relationships between inflammatory biomarkers and sensory responses, psychological factors and cervical extensor muscle fatty infiltrate in the acute and chronic stages of whiplash injury. 

## Materials and Methods

### Ethics Statement

Ethical clearance for this study was granted by the Medical Research Ethics Committee of The University of Queensland, Australia. All participants provided written informed consent.

### Study design

A prospective longitudinal design was used to study persons who sustained a whiplash injury from within three weeks of injury and followed to 3 months post-injury. An asymptomatic control group was assessed twice, a month apart. 

It has been consistently shown that recovery, if it takes place, will occur in the first 2-3 months post injury with a plateau of symptoms after this time [19,20]. For this reason our follow-up assessment point of 3 months is appropriate for the investigation of chronic WAD. 

### Participants

Forty-four volunteers (33 females, mean age 37.2 ± 8.9 years) reporting neck pain as a result of a motor vehicle crash (MVC) and 18 asymptomatic volunteers (14 females, mean age 40.1 ± 9.6 years) participated in the study. The participants with whiplash were recruited via local hospital emergency departments, primary care practices (medical and physiotherapy) and from print media advertisement. They were eligible if they met The Quebec Task Force Classification of WAD II (neck pain, limited range of movement and point tenderness in the neck) [21]. Subjects were excluded if they were WAD IV (fracture dislocation), WAD III (neurological deficit), experienced concussion, loss of consciousness or head injury as a result of the accident and if they reported a previous history of whiplash, neck pain or headaches that required treatment or if they had ever been diagnosed with tension-type headache or migraine. The asymptomatic control groups was recruited from the general community from print media advertisement and were included provided they had never experienced any prior pain or trauma to the cervical spine, head or upper quadrant that required treatment. Participants were also excluded if they had a previous diagnosis of a neurological disorder (eg multiple sclerosis), inflammatory disease (eg rheumatoid arthritis, lupus), metabolic disorders (eg diabetes), diagnosed cardiovascular disease (including hypertension), metastatic disease, disease processes that require ongoing management with steroids or NSAIDS, cigarette smoking, pregnancy or claustrophobia or an infection within the last 3 months as these factor may influence the measures to be collected. 

### Pain and disability

Participants were asked to rate their level of pain over the last 24 hours on a 10cm visual analogue (VAS) scale where 0=no pain and 10=worst pain imaginable. Pain related disability was measured using the Neck Disability Index which has been commonly used in whiplash research [22]. 

### Quality of Life

Quality of life was measured with the Short-Form 36 (SF-36) which provides an indicator across eight dimensions of health and well being: physical functioning, role limitations due to physical problems, bodily pain, general health perceptions, vitality, social functioning, role limitations due to emotional problems and mental health as well as a separate single item dimension called health transition [23]. Physical and mental component scores (PCS and MCS, respectively) are summary component scores of the eight dimensions [23]. 

### Posttraumatic stress symptoms

Posttraumatic stress symptoms were measured using the Posttraumatic Stress Diagnostic Scale (PDS)[24] a reliable self-report measure comprising 49 items with a short checklist identifying the traumatizing event. For the purpose of this study the index event was the accident associated with the neck injury. The cardinal symptoms of PTSD are rated on a four point scale of 17 items experienced in the past 30 days. Participants also rate the level of impairment caused by their symptoms across nine areas of life functioning. The total symptom severity scores was used.

### Pain catastrophising

Pain catastrophising was measured using the catastrophising subscale of the Coping Strategies Questionnaire (CSQ), a validated tool used to assess patient self-rated use of cognitive and behavioural strategies to cope with pain [25]. 

### Pain threshold measures

Pressure pain thresholds (PPT) were measured using a pressure algometer with a probe size of 1 cm^2^ and application rate of 40 kPa/s (Somedic AB, Farsta, Sweden) over the C5 spinous process and bilaterally over the muscle belly of tibialis anterior [26]. Participants were requested to push a button when the sensation changed from one of pressure alone to one of pressure and pain. Triplicate recordings were taken at each site and the mean values used for analysis.

Cold and heat pain thresholds were measured over the mid to lower regions of the cervical spine using the Thermotest system (SomedicAB, Farsta, Sweden). The thermode was preset to 30°C with the rate of temperature change being 1°C/s. Participants were asked to push a patient-controlled switch when the thermal sensation (cold or heat) first became painful [26]. Triplicate recordings were taken and the mean values used for analysis.

### Inflammatory biomarkers

Venous blood samples were collected by a qualified nurse. Blood samples were collected using a serum separator tube, allowed to clot for 30 min and then centrifuged for 15 min (1000 g). Serum samples were then stored at -80°C. ELISA assays for IL-1β, TNF-α and CRP were performed utilising commercially available kits (R&D Systems, Human IL-1 beta/IL-1F2 Quantikine HS ELISA Kit, HSLB00C).

### MRI muscle measures and analysis

Defined regions of interest (ROIs) were manually traced over each of the bilateral cervical extensor muscles (rectus capitis posterior minor, major, multifidii, semispinalis cervicis, capitis, splenius capitis and upper trapezius) on the axial T1-weighted images at each vertebral segment (C0–C7). The muscle fatty infiltrates (MFI) measures were created by taking a ratio between the pixel intensities of each muscle to that of a standardized region of intermuscular fat at the C2-level [13]. Histograms were created from each muscle ROIs with MRIcro software (www.mricro.com). All axial images were acquired on 256*256 pixel matrix with a 20x20 mm field of view (TR/TE: 448/14 ms). Slice thickness was 4 mm and a dedicated flexible neck coil was used as a receiver coil. A measure for total MFI was created and used for analyses by combining and averaging the MFIs for the extensor musculature bilaterally across all cervical segments (C0–7).

### Procedure

All measures were taken at both time points. The asymptomatic control participants did not complete the VAS of pain, NDI, PDS or CSQ questionnaires. One research assistant performed the physical measures and administered questionnaires on all participants at each assessment. The MRI measures were obtained immediately following collection of physical measures at the radiology centre by one investigator (JE) who was blind to the status of the participant in terms of questionnaire responses. The serum assays were performed by a research assistant blind to the nature of the participant (WAD or control) and to the results of any questionnaires or physical measures. 

### Sample size calculations

Based on pilot data of serum CRP from 10 patients with acute whiplash, the effect size for difference between those with higher (>5/10) levels of pain and those with lower pain levels (< 3/10) was 0.8. At 80% power and p=0.05, 18 participants/group were required. We accounted for 15% attrition and thus aimed to recruit 22 participants/WAD group. 

### Data analysis

WAD participants were classified into one of two groups based on results of the NDI at 3-6 months post-injury. The groups were recovered/mild disability (≤ 8-28% NDI) and ongoing moderate/severe pain and disability (≥ 30% NDI). These grouping criteria have been used extensively in our previous research of WAD [16,27,28] and recognise the need to consider the heterogeneous recovery of the condition [20]. Trajectory modelling analysis confirmed the validity of these NDI cut-off scores [20]. 

Data were analysed using SPPS (SPSS 20.0 for Windows, Chicago, Illinois, USA) software package. The inflammatory biomarker data were not normally distributed and underwent log transformation. A repeated measures analysis of variance (ANOVA) with a between subjects factor of Group (three levels: asymptomatic controls, recovered/ mild disability, moderate/severe disability) and a within subjects factor of Time (two levels: <1 month, 3 months post-injury) was performed. Differences between the groups were analysed with a priori contrasts. Significance was set at p < 0.05.Spearman two-tailed rank-order correlations were calculated at each of the two time points to assess relationship between variables. All values are given as median and 25%/75% percentile. A conservative significance was set at p<0.01 due to the number of analyses performed. 

## Results

### Participants

Four participants withdrew after the first assessment thus leaving 40 participants in the final analysis. Twenty participants were classified at 3 months as having moderate/severe disability (NDI≥ 30%) and 20 as being recovered or reporting only mild disability (≤ 28% NDI). Participant demographics are shown in Table 1. The groups were similar with respect to BMI. The WAD group with eventual moderate/severe disability, reported worse physical and mental health on the SF-36 than those with mild disability and controls and these were lower than Australian norms[29] . Forty-four percent of the participants with WAD experienced a rear end collision, 25% a front end collision; 13% a side impact collision;8% both front and rear collision and for 10% this data was not available. All participants with WAD reported neck pain, 76% reported headache and 54% associated arm pain. 

**Table 1:** Characteristics of participant groups at 3 months post injury.

Group	N	Age (years) (mean ± SD)	Gender (% female)	BMI (mean ± SD)	SF-36 Physical Health Summary Score	SF-36 Mental Health Summary score	NDI classification	NDI (mean ± SD)
Recovered/mild disability	20	34.9 (7.8)	70%	27.2 (6.2)	72 (17.4)	75.4 (21)	≤ 28%	7.1 (7.5)	
Moderate/severe disability	20	39.5 (9.5)	75%	26.5 (5.7)	46 (19.5)	46.3 (26)	≥ 30%	35.6 (13.2)	
Controls	18	40.1 (9.6)	77.7%	25.9 (6.4)	89 (6.7)	88.4 (11.6)			

NDI: Neck Disability Index; BMI: Body Mass Index; SF-36: Short Form 36

### Inflammatory biomarkers

The IL-1β data of 4 control participants and 3 WAD participants were below the limit of detection and were extrapolated beyond the lowest standard in the assay. 

#### TNF-α

There was a significant main effect for Group (F_2,53_=4.99, p=0.03) but no effect for Time (F_1,53_=0.004, p=0.95) and no Group x Time interaction (F_2,53_= 0.72, p=0.49). The recovered/mild disability WAD group had higher levels of serum TNF-α at both time points (p=0.04). Table 2

**Table 2:** Inflammatory marker concentrations in serum from asymptomatic controls and two whiplash groups (recovered or milder symptoms at 3 months) measured at two time points.

	Acute Stage WAD			Chronic Stage WAD (3 months)		
Inflammatory Marker Concentration (median, IQR)	Asymptomatic controls	Recovered or Mild WAD 0-28% NDI	Moderate/severe WAD ≥ 30% NDI	Asymptomatic controls	Recovered or Mild WAD 0-28% NDI	Moderate/severe WAD ≥ 30% NDI
TNF-*α* (pg/ml)	1.07 (0.56)	1.33 (0.88)*^	1.13 (0.65)	0.7 (0.13)	1.4 (0.94)* ^	0.99 (0.7)
IL-1B (pg/ml)	1.1 (0.9)	1.2 (0.27)	1.1 (0.7)	1.1 (0.6)	1.4 (0.8)	1.5 (0.3)
CRP (mg/l)	1.4 (0.25)	2.4 (0.32)*	2.5 (0.34)*	1.0 (0.13)	1.4 (0.37)	3.9 (0.37)* ^

NDI: Neck Disability Index; WAD: Whiplash Associated Disorders

* denotes significant difference from control group at p<0.05

^ denotes significant difference between WAD groups at p<0.05

#### IL-1β

There was no main effect for Group (F_1,53_=1.97, p=0.15) or Time (F_1,53_=1.96, p=0.17) nor any interaction effect between Group and Time (F_1,53_=1.25, p=0.29). Table 2

#### CRP

There was a significant main effect for Group (F_1,53_=3.99, p=0.04) and an interaction between group and Time (F_1,53_=5.24, p=0.02). At Time 1 (within 3 weeks of injury), the moderate/severe WAD group and recovered/mild WAD group both had higher levels of serum CRP compared to the control group (p<0.02) with no difference between the two WAD groups (p=0.88).

At Time 2 (3months post injury), the moderate/severe WAD group had higher CRP levels compared to the recovered/mild WAD group (p=0.02) and the control group (p=0.01). There was no difference between the recovered/mild WAD group and controls (p=0.07). Table 2

### Correlational analyses

Descriptive data for physical and psychological measures are provided in Table 3.

**Table 3:** Physical and psychological data (mean ± SD) for WAD groups at each time point.

	PPT (C5) kPa	PPT (Tibialis Anterior) kPa	Cold Pain threshold °C	Heat Pain threshold °C	Total MFI	PDS (symptom score)	Catastrophising subscale CSQ
< 3 weeks	193.5 (106.7)	382.8 (140.8)	13.6 (7.7)	43.5 (3.8)	0.2 (0.03)	12.9 (13)	7.1 (8.9)
> 3 months	245.3 (117.7)	421.4 (171.7)	12.8 (8.2)	43.9 (3.5)	0.2 (0.04)	10.7 (12.4)	6.6 (7.6)

PPT: pressure pain threshold; MFI: muscle fatty infiltrate; PDS: posttraumatic stress diagnostic scale; CSQ: coping strategies questionnaire

At Time 1 (within 3 weeks of injury) significant correlations were found between CRP serum levels and PPT at the cervical spine (r= -0.41, p=0.01), heat pain thresholds (r=-0.42, p=0.007) and cold pain thresholds (r=0.41, p=0.01). 

At Time 2 (3 months post injury) significant correlations were found between CRP serum levels and PPT at Tibialis Anterior (r=-0.55, p=0.001), and cold pain thresholds (r=0.42, p=0.01). There was also a significant negative correlation between serum TNF-α and Total MFI (r=-0.51, p=0.001). 

No significant correlations were found between serum biomarker levels and symptoms of PTSD (PDS total symptom score) or pain catastrophising scores on the CSQ. 

## Discussion

Despite whiplash injury being of traumatic nature in onset, there are few studies available that have prospectively investigated inflammatory processes in this condition. The results of our study showed differences in levels of certain inflammatory biomarkers between those who recover well following the injury compared to individuals who develop chronic moderate to severe pain related disability. We also demonstrated consistent moderate relationships between serum inflammatory biomarker levels and measures of hyperalgesia and muscle morphology but no relationship with posttraumatic stress symptoms or catastrophising. 

At the baseline time point within 2-3 weeks post injury both WAD groups showed elevated serum levels of CRP compared to controls. CRP is an acute phase marker of low-grade inflammation [5] and the elevation of CRP levels seen in our study may be caused by inflammation of injured local soft tissues in the cervical spine. By 3 months post injury CRP levels in recovered participants and those with only mild disability were no different from controls but remained elevated in participants with poor functional recovery indicating ongoing low-grade inflammation in this group. This may be due to ongoing inflammation from injured soft tissues that are yet to heal and support for this scenario can be found in clinical [30] and cadaveric [31] studies where there is evidence of unresolved tissue lesions present in chronic WAD. CRP levels in the group with poor recovery were similar to those reported for other painful musculoskeletal conditions including work related musculoskeletal disorders [5] and acute sciatic pain [32]. Elevated CRP levels are also associated with systemic diseases including cardiovascular disease, metabolic disease and diabetes [5]. However we excluded participants from our study if they had been diagnosed with such conditions and also limited the upper age limit to 55 years in order to decrease the likelihood of undiagnosed cardiovascular diseases. Thus it is unlikely that our participants suffered such conditions. 

CRP levels showed moderate correlations with some sensory measures including mechanical hyperalgesia (lowered PPTs) both locally over the cervical spine and at a remote lower limb location as well as with thermal (heat and cold) hyperalgesia. Associations with cold pain thresholds were found at both the acute and chronic assessment time points. The presence of widespread sensory hyperalgesia is a commonly found feature of WAD [33,34] with some measures particularly cold hyperalgesia having moderate evidence as a prognostic indicator of poor recovery [35]. It is generally considered that this sensory hypersensitivity represents augmented or hyperexcitable central nervous system nociceptive processing [34,36] but mechanisms contributing to the central hyperexcitability are not clear. The results of our study suggest that persistent low grade systemic inflammation may also be an explanation for the presence of these phenomena. The relationship of CRP with the sensory features of WAD and the persistence of increased levels from the acute to chronic stage of the condition in those with poor recovery suggests that CRP may contribute to the initiation and maintenance of chronic whiplash pain. There is also evidence available to indicate that elevated levels of CRP in generally healthy people are associated with higher pain sensitivity [37]. This may lead to an increased vulnerability for the development of chronic pain and we cannot rule out that the WAD participants in our study with poor recovery may have had pre-existing elevated CRP levels rather than the elevation being as a result of the injury. 

There was no difference between the WAD groups and healthy controls at either time point in serum levels of IL-1β. Findings of elevated serum levels of IL-1β in musculoskeletal pain conditions are inconsistent, with some studies showing elevated levels compared to controls in overuse upper limb disorders [38] and others, for example carpal tunnel syndrome [39], showing no difference. Overuse upper limb disorders likely involve more injured sites than either WAD or carpal tunnel syndrome resulting in greater concentrations of serum IL-1β. Additionally, increased levels of IL-1β have been found in local structures such as the intervertebral disc in patients with low back pain [40] and in animal models of this condition [41] but not in serum of patients with low back pain [5,42]. It is possible that there are insufficient quantities of IL-1β in the serum of patients with WAD but this would not preclude the possibility of increased levels in local cervical injured tissues. 

At both the acute time point (2-3 weeks post injury) and at the chronic stage of the injury (3 months) serum TNF-*α* levels were significantly higher in the recovered/mild disability group compared to healthy controls and those with eventual moderate/severe pain related disability. This apparently paradoxical finding of greater serum TNF-*α* levels in those with good or fair recovery was at odds with our hypothesis and with investigation of other musculoskeletal conditions such as upper extremity overuse injuries[38] and chronic low back pain [43] where increased levels of serum TNF-α have been found and shown to be positively associated with disability levels [38] . Whilst the presence of increased levels of pro-inflammatory cytokines, including TNF-*α* are generally considered to be associated with adverse outcomes following trauma [44], some data indicate the opposite. In both human and animal studies, Namas and colleagues [44] have demonstrated that an early robust TNF-α response is associated with survival following trauma. These studies involved more severe traumatic injuries than those sustained by whiplash injury but it could be speculated that increased levels of TNF-α may also be associated with better recovery and this requires further investigation. 

The results of our study showed greater differences for CRP between the WAD groups and controls than either of the two cytokines measured. These results are consistent with other studies of musculoskeletal pain conditions where stronger associations with CRP and functional impairment have been found [38] as well as relationships with sensory measures [37]. This may reflect less sensitivity of detection of IL-1β and TNF-α in serum [38] or may indicate relatively greater levels of CRP and this requires further investigation. 

Our data also demonstrated an inverse moderate relationship between serum TNF-*α* levels and fatty muscle infiltrate identified on MRI. This finding does not support our hypothesis that increased circulating levels of inflammatory biomarkers may contribute to the presence of fat in the cervical muscles of patients with WAD. It has long been recognised that TNF-*α* is associated with muscle degradation and loss of muscle mass in inflammatory conditions such as cancer, AIDS and chronic obstructive pulmonary disease [45] but the levels of TNF-*α* are much higher than those found in our study. Paradoxically, there is also evidence available from animal studies indicating that TNF-*α* is involved in the recovery of muscle function after muscle injury [46] and in a human study of chronic obstructive pulmonary disease, levels of quadriceps TNF-*α* correlated positively with muscle function[47]. These findings have led to a proposal that local inflammation may not be detrimental but even helpful in maintaining muscle integrity and function[17]. It is possible that the higher levels of TNF-*α* in our recovered/milder disability group may have facilitated muscle recovery. 

We found no association between levels of inflammatory biomarkers and scores on the psychological questionnaires. Several lines of evidence indicate that chronic PTSD is associated with increased levels of cytokines and to a lesser extent CRP [12]. Most of these studies have included patients with a primary diagnosis of PTSD and/or comorbid depression [12] that likely had more severe PTSD symptomatology than the whiplash injured participants of our study. None of our participants had been formally diagnosed with PTSD via structured interview with a psychologist and only 20% had a probable diagnosis of PTSD at 3 months based on the criteria of the PDS. Thus the lower severity levels of our sample may be one reason for the lack of association found between inflammatory biomarkers and PTSD symptoms. Preliminary data also point to a relationship between pain catastrophising and inflammation. Edwards et al [13] demonstrated in healthy controls that higher levels of catastrophising were associated with increased serum levels of interleukin-6 after the administration of a noxious stimulus. We found no relationship between pain catastrophising and CRP in either the acute or chronic stage of injury. Our findings indicate that inflammatory biomarker levels may be more associated with pain and disability than more specific psychological constructs. 

Although the prescription of anti-inflammatory medication is advocated for acute musculoskeletal pain [48] and is commonly used in the management of WAD [49], there are few available trials demonstrating evidence for such medications. The results of our study would suggest that non-steroidal anti-inflammatories may be beneficial for this condition. The early presence of hyperalgesia, particularly cold hyperalgesia is a consistent adverse prognostic indicator for poor recovery [35] and its consistent and moderate association with CRP found in this study suggest that decreasing levels of CRP may attenuate processes underlying hyperalgesia and potentially improve recovery following whiplash injury. 

There are limitations to our study. Participants were requested to refrain from taking anti-inflammatory medications 7 days prior to testing. Whilst most reported adherence with this request, some (19%) failed to do so and this may have influenced our results. In particular IL-1B can be reduced by low dose aspirin[50] and this may have contributed to the lack of significant differences found in this marker. Additionally, the BMI data of our participants indicate that they were in the overweight range. However this applied to the control group as well and therefore is unlikely to explain the difference in inflammatory marker levels. 

In summary, the results demonstrate initially higher levels of serum CRP following whiplash injury that persist in those with persistent moderate/severe pain and disability and show moderate associations with mechanical and cold hyperalgesia. In contrast serum levels of TNF-α are elevated in those with good or fair recovery and are negatively associated with amounts of fatty infiltrate in the cervical extensor muscles. Inflammatory biomarkers appear to be associated with the presentation of acute and chronic WAD. 
